# Combination treatment with artemisinin and oxaliplatin inhibits tumorigenesis in esophageal cancer EC109 cell through Wnt/β‐catenin signaling pathway

**DOI:** 10.1111/1759-7714.13570

**Published:** 2020-07-12

**Authors:** Tao Wang, Jian Wang, Wei Ren, Zhu‐Long Liu, Yu‐Feng Cheng, Xiao‐Mei Zhang

**Affiliations:** ^1^ Department of Radiotherapy, Qilu Hospital, Cheeloo College of Medicine Shandong University Jinan China; ^2^ Department of Ultrasound Shandong Province Coal Taishan Sanatorium Taian China; ^3^ Department of Radiotherapy The People's Hospital of Lanling County Linyi China

**Keywords:** Artemisinin, cell proliferation, esophageal cancer, Oxaliplatin, Wnt/β‐catenin

## Abstract

**Background:**

Esophageal cancer (EC) is a prevalent malignant cancer worldwide. Interestingly, the antimalaria compound artemisinin (ART) is also reported to have anticancer potential, although its underlying mechanism in EC is unclear. In this study, we explored the anticancer role of ART in EC109 and further explored the combination of ART and oxaliplatin (OXA) for their synergetic anticancer functions.

**Methods:**

Human EC cell line EC109 was used. After ART or oxaliplatin (OXA) treatment, cell proliferation, migration, and invasion were measured by MTT, transwell, and scratch wound assays, respectively. Flow cytometry was performed to examine the cell cycle and apoptosis. The mRNA and protein levels were determined using qRT‐PCR and western blotting.

**Results:**

The migration and invasion abilities of EC109 were suppressed by ART. This was due to the inhibitory effect of ART on the Wnt/β‐catenin signaling pathway. The levels of β‐catenin, c‐myc, and survivin were also downregulated by ART. ART inhibits the proliferation of EC109 cells by arresting the cells in the G1‐phase of cell cycle. By using LiCl, an activator of the Wnt/β‐catenin pathway, we further verified that the inhibition of the Wnt/β‐catenin pathway was indeed due to ART. Remarkably, ART enhanced the anticancer effects of OXA in EC109 cells. OXA combined with ART was found to be more efficient in decreasing tumor growth compared to the individual drugs.

**Conclusions:**

ART could suppress tumor progression by inhibiting Wnt/β‐catenin signaling pathway, and it may also enhance the antitumor effect of OXA in EC. Thus, ART could be a novel anticancer drug for EC treatment.

**Key points:**

**Significant findings of the study:**

ART could be a novel anticancer drug for esophageal cancer (EC) treatment.

**What this study adds:**

Combination treatment with artemisinin and oxaliplatin inhibits tumorigenesis in esophageal cancer EC109 cells through the Wnt/β‐catenin signaling pathway.

## Graphical abstract

Artemisinin (ART) inhibited cell proliferation, migration, and invasion but promoted apoptosis in esophageal cancer EC109 cells. These effects were facilitated by the inhibition of the Wnt/β‐catenin signaling pathway. Furthermore, ART also enhanced the antitumor effect of oxaliplatin (OXA) in esophageal cancer cells.

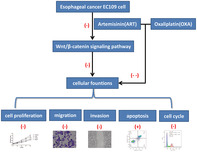

## Introduction

Esophageal cancer (EC) is one of the most common causes of cancer mortality in the world.^1^ Although the treatment of EC has been greatly improved in recent years, the prognosis of EC remains unsatisfactory.^2^ The present treatment for EC includes surgery, chemotherapy, and radiotherapy.^3^ Chemotherapy is the key treatment for metastatic diseases which can improve quality of life.^4^ As there are no easily distinguishable symptoms, the early prognosis of EC is difficult and at the time of diagnosis, many patients are already in the advanced stages.^5^ Therefore, it is crucial to seek an effective treatment method that can improve the quality of life along with the survival time of EC patients.

A previous study suggests that the overall survival rate in cancer patients can be improved using broadly effective phytochemicals that inhibit metastasis and invasion with tolerable side effects.^6^ Artemisinin (ART) is a sesquiterpene lactone isolated from the Chinese plant *Artemisia annua* (commonly known as qinghaosu or sweet wormwood) and has been used since 1970.^7^ Presently, ART and its derivatives have been identified as the most effective drugs to treat chloroquine‐resistant malaria without the notable side effects.^8^, ^9^ In addition to the antimalarial properties, ART is also reported to exhibit an antitumor function.^10^, ^11^, ^12^

Wnt/β‐catenin is a powerful signaling pathway that plays a crucial role in cell fate determination, survival, and proliferation in multiple tissues.^13^ Like many other cancers, the occurrence and progress of EC is also closely related to the activation of oncogenic signaling pathways, and inactivation of tumor suppressor signaling pathways.^14^ Specifically, the misregulation of the Wnt/β‐catenin signaling pathway mediated by the tumor suppressor or activating agents has been associated with EC.^15^, ^16^ Interestingly, several studies have suggested that ART imparts tumor attenuation through the Wnt/β‐catenin signaling pathway.^17^, ^18^ However, its exact role in regulating the Wnt/β‐catenin pathway in EC is unclear.

Oxaliplatin (OXA), a platinum‐based chemotherapeutic agent with a 1,2‐ diaminocyclohexane carrier ligand, has shown efficacy against many tumor cells, and possess no cross‐resistance with cisplatin and carboplatin.^19^, ^20^ OXA can also be used as an ideal chemotherapy drug for the treatment of esophageal related cancers but has limited effect in the single‐drug therapy.^21^ Despite the initial efficiency, most anticancer drugs eventually develop chemoresistance in nearly all metastatic patients. This is the major reason for the failure of chemotherapy.^22^ OXA is widely used in combination therapies with other anticancer drugs such as 5‐fluorouracil, leucovorin, irinotecan, and folinic acid.^23^, ^24^ However, the combined efficacy of ART and OXA in EC is unknown.

Therefore, in this study, we first tested whether ART interfered with EC tumor growth by blocking the unrestricted activation of the Wnt/β‐catenin signaling pathway. Further, we tested for the additive effects of OXA and ART against EC.

## Methods

### Cell cultures and material

The human EC cell line EC109 was obtained from Cell Bank of Chinese Academy of Sciences, Shanghai, China. The cells were cultured in Dulbecco's Modified Eagle Medium (DMEM, Gibco, USA) with 10% FBS and 1% streptomycin/penicillin at 37°C in a 5% CO_2_ incubator. Artemisinin (ART), oxaliplatin (OXA), and LiCl were purchased from Sigma‐Aldrich (Shanghai, China). ART and OXA were dissolved in dimethyl sulfoxide (DMSO; Sigma, USA) and added to 2 mg/mL phosphate‐buffered saline (PBS), used as a storage solution. The solution was then added into the cell culture medium at various concentrations. The final concentration of DMSO was <0.1% (v/v) in all experiments.

### MTT assay

5‐Diphenyltetrazolium bromide (MTT) (Sigma‐Aldrich) assay was performed to measure cell proliferation. EC109 cells (2 × 10^4^ cells/mL) were cultured in 96‐well plates with different doses of ART and OXA. After the drug treatment, 0.5 mg/mL MTT was added into each well at 24, 48, 72, and 96 hours and cells were further incubated for 4 hours at room temperature (RT). The supernatants were then discarded and colored formazan crystals were dissolved with 150 μL/well of DMSO. Further, cells were treated with ART and/or OXA (from 0–100 μM) to generate curves and calculate half‐maximal inhibitory concentration (IC50) values. A microplate reader (Bio‐Rad, USA) at 570 nm was used to analyze the OD values.

### Scratch wound healing assay

Scratch wound healing assay was used to detect cell migration. EC109 cells were seeded into six‐well plates and treated with 0, 5, 10, or 20 μM ART and/or OXA. A wound was introduced to the cell layers using a 200 μL pipette tip and cells were cultured in 10% FBS‐supplemented DMEM medium. Cell migration was measured at 0 and 48 hours with an inverted microscope at 100× magnification.

### Transwell assay

Transwell chambers (Corning, USA) were used to detect cell invasion. Briefly, 200 μL of EC109 cells (1 × 10^5^ cells) treated with 0, 5, 10, or 20 μM ART and/or OXA were added in the upper chamber of a transwell apparatus coated with Matrigel (Corning, USA) and incubated in DMEM with 10% FBS for 48 hours. Cells that had migrated to the lower chamber were fixed for 20 minutes in 1% formaldehyde and stained for 20 minutes in crystal violet (0.1%). Stained cells were visualized with a microscope (Olympus) and five randomly selected fields were used to count the number of invaded cells.

### Cell apoptosis analysis

The Annexin V‐FITC kit (Biosea Biotechnology Co., Beijing, China) was used to test cell apoptosis. EC109 cells (5.0 × 10^5^ cells/mL) were treated with 0, 5, 10, or 20 μM ART and/or OXA and resuspended in PBS buffer. Subsequently, the cells were double‐stained using Annexin V‐Alexa Fluor 647 and propidium iodide (PI). Finally, the apoptotic rate was analyzed using a flow cytometer (BD Biosciences, San Diego, CA, USA).

### Cell cycle analysis

EC109 cells (1 × 10^6^ cells/mL) were treated with ART and/or OXA for up to 48 hours. For cell cycle analysis, cells were harvested, fixed with 70% cold ethanol, incubated with RNase, and stained with propidium iodide. The cell cycle was detected using flow cytometry (BD Biosciences) and data was analyzed using FlowJo v7.6 software (version 3.2, Verity Software House, USA).

### Quantitative real‐time PCR (qRT‐PCR) assays

Total RNA from EC109 cells was extracted using TRIZOL reagent (Invitrogen). Sample concentrations were measured using a NanoDrop ND‐1000 spectrophotometer (NanoDrop, USA). Then, total RNA (500 ng) was reverse‐transcribed into cDNA using the PrimeScript RT reagent Kit (TaKaRa, Dalian, China) and analyzed by qRT‐PCR using a SYBR Green PCR kit (TaKaRa). The sequences of the used primers were as following: c‐myc: forward: 5′‐CCCAGCGAGGACATCTGGAAGAA‐3′, reverse: 5′‐GAGAAGCCGCTCCACATGCAGTC‐3′; survivin: forward: 5′‐AGCCCTTTCTCAAGGACCAC‐3′, reverse: 5′‐GCACTTTCTTCGCAGTTTCC‐3′; β‐actin forward primer: 5′‐ACACTGTGTGCCCATCTACGAGG‐3′, reverse primer: 5′‐AGGGGCCGGACTCGTCGTCATACT‐3′. The primers were obtained from GenePharma (GenePharma, Shanghai, China). The β‐actin was used to normalize the transcript levels of mRNA. Relative expression was calculated using the 2^‐ΔΔCt^ method.^25^

### Western blot analysis

Total protein was extracted from EC109 cells using RIPA lysis buffer (Sigma, USA). The protein sample (50 μg per sample) was separated on a 10% SDS‐PAGE gel and transferred onto a polyvinylidene difluoride membrane. These membranes were blocked with 5% nonfat milk for 2 hours and incubated overnight with primary antibodies anti‐β‐actin (1:1000, ab179467, Abcam, UK), anti‐c‐myc (1:1000, ab32072, Abcam, UK), antisurvivin (1:1000, ab76424, Abcam, UK) at 4°C. After washing three times, the membranes were incubated with a peroxidase‐labeled secondary antibody (anti‐rabbit IgG, 1:2000, ab6721, Abcam, UK) for 2 hours. Enhanced chemiluminescence (ECL) (ThermoFisher, USA) was used to visualize the protein bands, and analysis was carried out using the Image Laboratory Software (Bio‐Rad, USA).

### Statistical analysis

All data represent the mean ± SD from at least three independent experiments. A Student's *t*‐test was used to identify significant differences between two groups and a one‐way ANOVA with Tukey's post hoc test was used to compare the means of more than two groups. All statistical analyses were performed using SPSS 22.0 (Chicago, IL, USA) and GraphPad Prism 7.0 (GraphPad, San Diego, CA, USA). *P* < 0.05 was considered statistically significant.

## Results

### Artemisinin inhibits the malignant progression of EC cells

**Figure 1:**
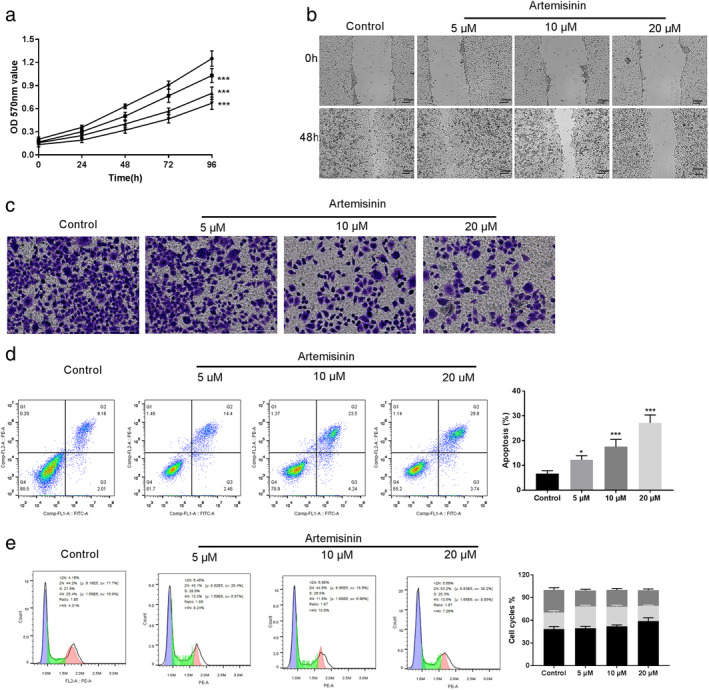
Artemisinin (ART) inhibits the malignant progression of EC109 cells. (**a**) Relative cell proliferation of EC109 treated with ART (0–20 μM) by MTT assay (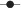) Control (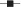) 5 μM (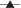) 10 μM (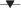) 20 μM. (**b**) Representative photomicrographs of initial and final wounds at 0 and 48 hours are shown at 100× magnification by scratch wound healing assay. (**c**) Transwell assay shows the invasion of EC109 cells. (**d**) Effect of ART on EC109 cell apoptosis by flow cytometry. (**e**) Result of cell cycle of EC109 cells after treatment with ART by flow cytometry. Each group represented the mean ± standard deviation (SD) of at least three independent experiments. **P < 0.01, and ***P < 0.001 versus control group (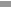) G2/M (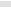) S (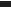) G0/G1.

First, an MTT assay was conducted to investigate the effects of ART on the cell fate of EC109 cells (Fig 1a). Compared to the control group, artemisinin (ART, 0–20 μM) treatment significantly suppressed the cell viabilities (*P* < 0.001). Further, using the wound healing assay, migration in EC109 cells was evaluated. Here too, ART significantly inhibited the cell migration in a dose‐dependent manner, and 20 μM of ART was most effective in migration suppression compared with the control group (Fig 1b). Additionally, a transwell assay was used to explore the invasive ability of EC109 cells. Compared with the control group where abundant cells translocated to the underside well, a dose‐dependent decrease in cell invasion activities was noted upon treatment with 5, 10, and 20 μM of ART (Fig 1c). Furthermore, flow cytometry assay was conducted to measure the apoptosis and cell cycle of ART‐treated EC109 cells. As shown in Fig 1d, apoptosis in EC109 cells significantly increased with the increasing concentrations of ART compared to the control group (*P* < 0.001). Furthermore, an increase in concentrations of ART significantly accumulated the EC109 cells arrested in the G0/G1 phase. Especially, at 20 μM ART, a significant number of 58.89% ± 4.50% (*P* < 0.001) tumor cells were in the G0/G1 phase (Fig 1e). Therefore, these findings suggest that artemisinin increased apoptosis, but inhibited the proliferation, migration, and invasion of EC109 cells.

### Artemisinin inhibits Wnt/β‐catenin signaling pathway

**Figure 2:**
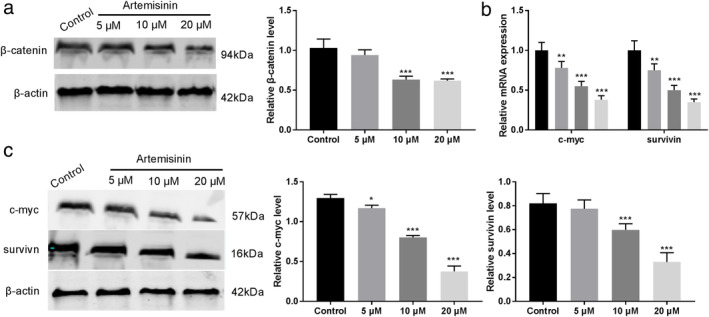
Artemisinin (ART) inhibits the Wnt/β‐catenin signaling pathway. (**a**) β‐catenin expression was downregulated in EC109 cells measured by western blot. (**b**) qRT‐PCR was used to measure the expression of c‐myc and survivin in EC109 cells treated with ART (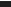) Control (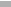) 5 μM (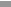) 10 μM (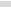) 20 μM. (**c**) Western blot analysis was performed for c‐myc and survivin. Data shown are the means ± SD of at least three independent experiments. *P < 0.05, **P < 0.01, ***P < 0.001 versus control group.

To determine whether ART treatment affected the Wnt/β‐catenin signaling pathway in EC109 cells, the key proteins of the Wnt/β‐catenin pathway were analyzed by western blot. The results showed that the expression of β‐catenin had the greatest reduction with the increase of ART concentration (*P* < 0.001) (Fig 2a). Further, we tested if the ART effect on β‐catenin could inhibit the transcription of the target genes of Wnt/β‐catenin (c‐myc and survivin). The qRT‐PCR and western blot analysis showed a decrease in mRNA and protein levels of c‐myc and survivin. This effect was also apparent in a concentration‐dependent manner upon treating with different concentrations of ART (*P* < 0.001) (Fig 2b,c).

### Artemisinin could suppress proliferation and metastasis of EC109 cells partially depending on the Wnt/β‐catenin pathway

**Figure 3:**
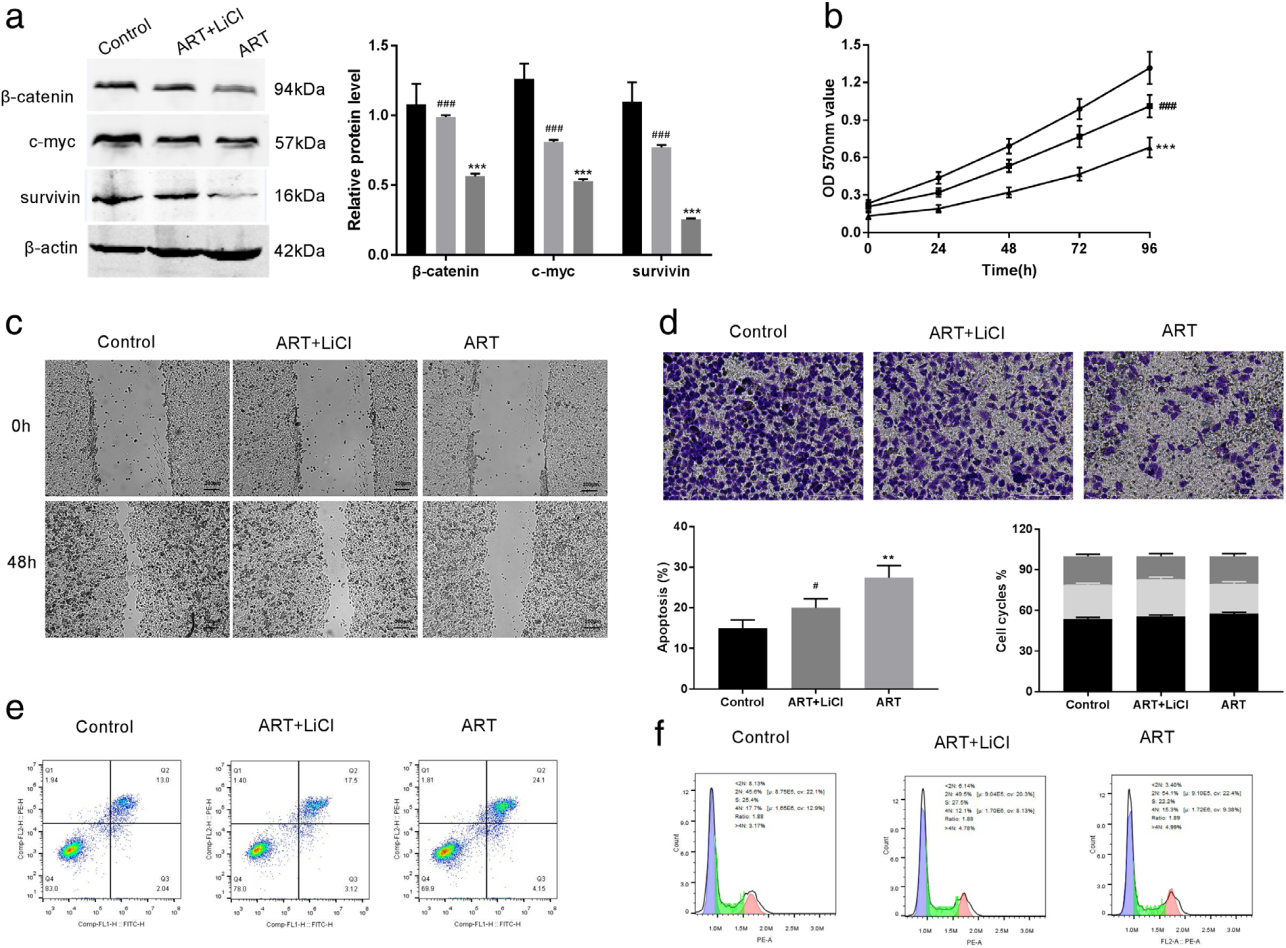
Artemisinin (ART) could suppress the proliferation and metastasis of EC109 cells depending on the Wnt/β‐catenin pathway. (**a**) Upon LiCl treatment, the increased protein level of β‐catenin, c‐myc, and survivin caused by ART was attenuated. (**b**) Cell proliferation impaired by the ART was drastically recovered (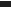) Control (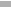) ART + LiCl (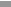) ART. (**c,d**) The migration and invasive abilities of the ART‐treated cells were also recovered. (**e**) Apoptosis in the ART‐treated cells decreased (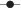) G2/M (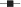) S (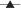) G0/G1. (**f**) The cell cycle arrest in the ART‐treated was also recovered. All results are presented as the mean ± SD of at least three independent experiments. ***P < 0.001 versus Control group, ^*###*^ *P* < 0.001 versus ART group.

To verify whether the aforementioned antitumor effects of ART were indeed associated with the Wnt/β‐catenin signaling pathway, ART (20 μM) treated cells were subjected to 20 mM LiCl, an activator of the Wnt/β‐catenin signaling pathway. Western blot assay demonstrated that ART mediated downregulation in the protein levels of β‐catenin, c‐myc, and survivin was attenuated upon treatment with LiCl (*P* < 0.001) (Fig 3a). Intriguingly, LiCl treatment also partially alleviated the inhibition of the cell proliferation caused by ART (*P* < 0.001) (Fig 3b). Moreover, ART mediated downregulation of cell migration and invasion was also recovered by LiCl treatment (Fig 3c,d). Also, cell flow cytometry assay elucidated that LiCl reduced the ART mediated apoptosis and decreased the cell arrest in the G0/G1 phase in EC109 cells (Fig 3e,f). Overall, these results strongly indicate the involvement of the Wnt/β‐catenin signaling pathway in ART‐mediated inhibition of EC progression.

### Combination of artemisinin and oxaliplatin may reduce chemoresistance in EC109 cells

**Figure 4:**
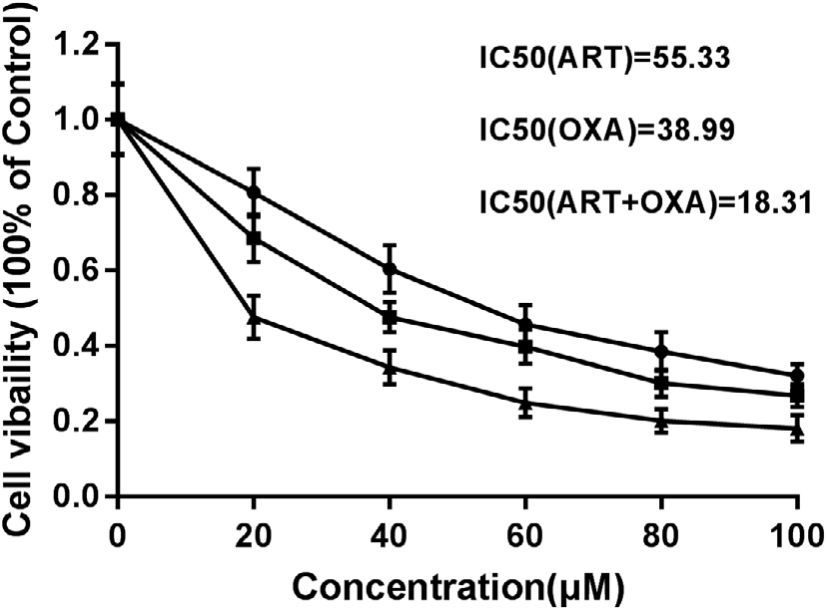
Artemisinin (ART) and oxaliplatin (OXA) reduces chemoresistance in EC109 cells. IC50 of ART, OXA, and the combination of ART and OXA were determined by treating parental EC109 cells in a dose‐dependent manner. The data are presented as the mean ± SD of at least three independent experiments (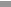) ART (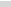) OXA (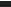) ART+OXA.

OXA, a chemotherapeutic drug is also used for the treatment of EC. We speculated that a combination of OXA and ART could improve the overall efficacy. Also, by reducing the amount of OXA in combination therapy compared to the single drug treatment might reduce the side effects of large doses. A MTT assay was conducted to investigate the effects of ART and OXA on the cell fate of EC109 cells. We found that after treating cells with ART, OXA, or ART+OXA (0–100 μM) for 48 hours, cell viabilities were significantly suppressed. Additionally, the efficiency of the cell inhibition was in the following order: ART+OXA > OXA > ART. The half‐maximal inhibitory concentration (IC50) of ART and OXA alone were 55.33 μM and 38.99 μM respectively. Interestingly, the IC50 of the combination (ART+OXA) was 18.31 μM (Fig 4).

### Artemisinin enhances the antitumor effects of oxaliplatin in EC109 cells

**Figure 5:**
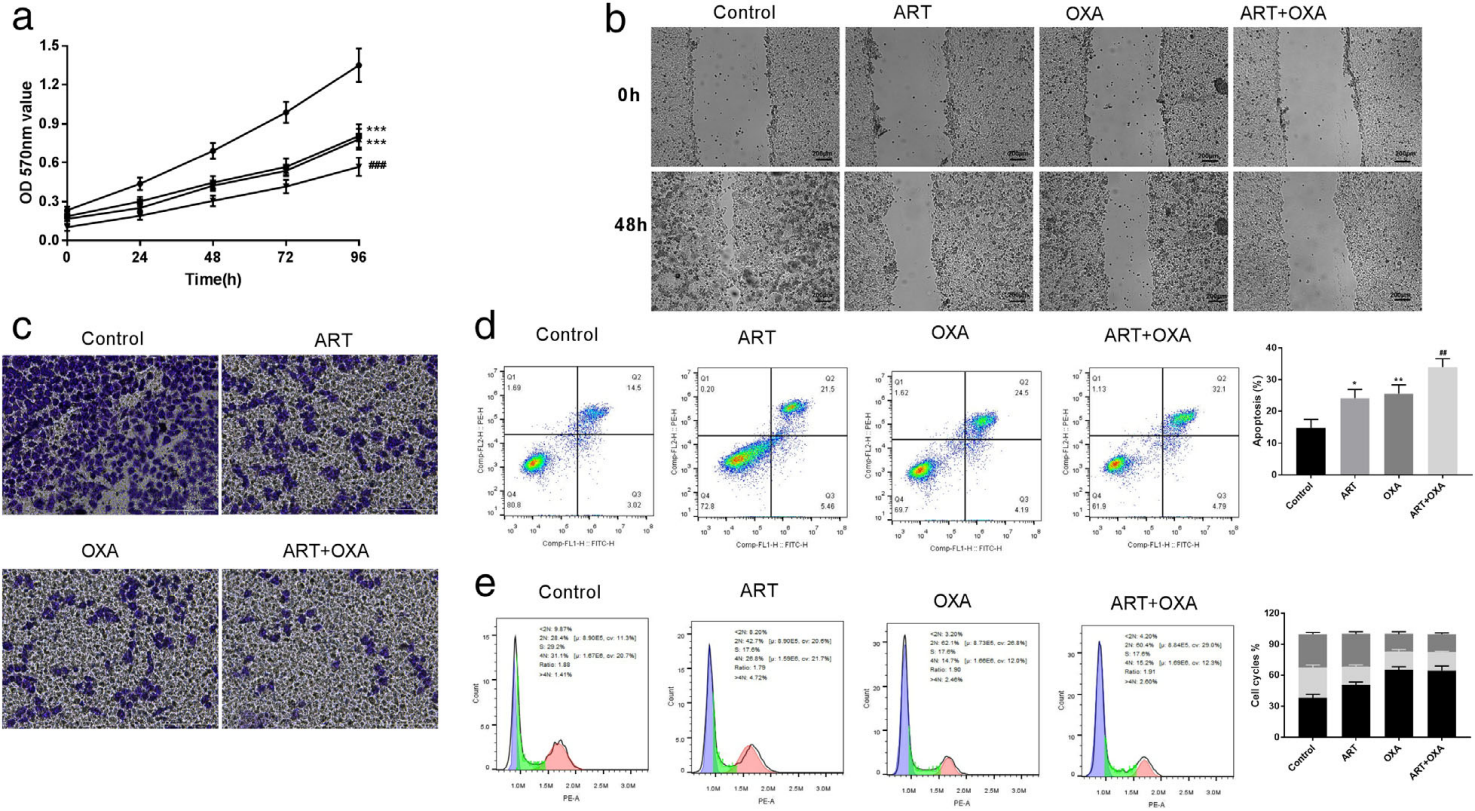
Artemisinin (ART) enhances the antitumor effects of oxaliplatin (OXA) in EC109 cells. (**a**) An MTT assay showing the proliferation of EC cells treated with ART (20 μM) and/or OXA (18 μM) (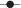) Control (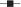) ART (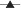) OXA (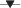) ART+OXA. (**b**) Scratch wound healing assay showing the effect of ART and/or OXA on the migration of EC109 cells. (**c**) Transwell assay showing the effects of ART and/or OXA on the invasion of EC109 cells. (**d**) Effect of ART on EC109 cell apoptosis using flow cytometry. (**e**) Result of cell cycle of EC109 cells after treatment with ART using flow cytometry (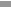) G2/M (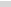) S (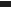) G0/G1. Each group represented the mean ± standard deviation (SD) of at least three independent experiments. *P < 0.05, **P < 0.01, and ***P < 0.001 versus control group. ^##^ *P* < 0.01, ^###^ *P* < 0.001 versus ART or OXA group.

To further verify the combined effect of ART and OXA, we selected 20 μM ART and 18 μM OXA for the following experiments. MTT assay showed that cell proliferation against the ART+ OXA combination was significantly suppressed compared to the ART or OXA alone (*P* < 0.001) (Fig 5a). Similarly, in the wound healing assay, a combination of ART and OXA reduced cell migration more efficiently compared to either drug alone (Fig 5b). Also in the transwell assay, the effect of the combination of ART and OXA in reducing cell invasion was more pronounced compared to either ART or OXA (Fig 5c). Furthermore, when flow cytometry analysis was performed to determine the combined effect of ART and OXA on cell apoptosis, the apoptosis rate for the combination of ART + OXA was significantly higher compared to the individual drugs (*P* < 0.01) (Fig 5d). Similarly, greater accumulation of the cells arrested in the G0/G1 phase was observed for the combination than the ART or OXA treated cells (Fig 5e).

## Discussion

There is a high incidence of EC in China.^26^ Despite recent advances in the treatment of EC, the prognosis for EC remains poor.^27^ Several studies have shown the potential antitumor therapeutic effects of ART both in vitro and in vivo.^17^, ^18^ In this study, we assessed the efficacy of ART against EC cell line EC109. We found that ART suppressed the growth and promoted cell apoptosis in a dose‐dependent manner. Furthermore, wound healing and transwell assays also revealed the ART mediated dose‐dependent decrease in cell migration and invasion. More strikingly, cell cycle arrest of EC109 cells was also observed upon treatment with ART. In total, these results strongly suggest that ART could be a potential chemopreventive agent for EC.

The Wnt/β‐catenin pathway plays a critical role in every stage of cancer progression, including cell proliferation, development, and metastasis.^15^, ^16^, ^28^, ^29^ Furthermore, the Wnt/β‐catenin signaling pathway can be effectively inhibited by ART.^17^, ^18^, ^30^ In this study, we also observed the inhibitory effect of ART on the hyperactive Wnt/β‐catenin signaling pathway. The Wnt/β‐catenin target genes, C‐myc and survivin, are reported to be upregulated in EC,^31^ and a decrease in expression of these genes promote cell apoptosis and inhibits cell division.^32^ Importantly, ART treatment significantly decreased the levels of β‐catenin, c‐myc, and survivin. Furthermore, the experiments were also carried out using the Wnt/β‐catenin signaling pathway activator LiCl^15^ in combination with ART. The results from these experiments strongly suggested that inhibition of the Wnt/β‐catenin signaling pathway was indeed mediated by ART. Overall, these findings suggest that ART may impede the development of EC through the Wnt/β‐catenin signaling pathway.

Chemotherapy is a double‐edged sword, along with antitumor effectiveness; it can simultaneously lead to several side effects such as diarrhea, headache, and ototoxicity.^33^, ^34^ In the combination chemotherapy treating esophageal squamous carcinoma, OXA is more effective but less toxic than cisplatin.^4^ However, its wider application is still limited by the side‐effects and drug resistance. Therefore, in this study, we explored the combinational antimetastatic effect of OXA with ART. Interestingly, in comparison to the individual drugs, the combination of ART and OXA was more efficient in inhibiting the cell proliferation, migration, and invasion in the EC109 cells. This strongly suggests that ART could also enhance the antitumor effects of OXA in EC.

In conclusion, our results showed that artemisinin inhibited cell proliferation, migration, and invasion but promoted apoptosis in EC109 cells. These effects were facilitated by the inhibition of the Wnt/β‐catenin signaling pathway. Furthermore, ART also enhanced the antitumor effect of OXA in EC. Although this study was limited to EC109 cells, in future more EC cell lines should be investigated to strengthen these findings. Nevertheless, these results significantly suggest that ART together with OXA can be used for the clinical treatment of EC.

## Disclosure

The authors have no conflicts of interest to report.
